# Segmented MR Images by RG-FCM subjected to Non-Uniform Compression comprising Cascade of different Encoders

**DOI:** 10.2174/0115734056356911250220124124

**Published:** 2025-03-17

**Authors:** Lovepreet Singh Brar, Sunil Agrawal, Jaget Singh, Ayush Dogra

**Affiliations:** 1 Department of Electronics and Communication Engineering, University Institute of Engineering and Technology, Panjab University, Chandigarh 160014, India; 2 Chitkara University Institute of Engineering and Technology, Chitkara University, Punjab, 140401, India

**Keywords:** Magnetic Resonance Imaging, Informative part, machine learning-based segmentation, Set-partitioning in Hierarchical trees (SPIHT), Huffman encoding, Compression Ratio (CR)

## Abstract

**Introduction:**

The fundamental problem with the transmission and storage of medical images is their inherent redundancy and large size necessitating higher bandwidth and a significant amount of storage space.

**Objectives:**

The main objective is to enhance the compression efficiency through accurate segmentation followed by non-uniform compression through a cascade of encoders.

**Background:**

Due to a sharp growth in digital imaging data, it is highly desirable to reduce the size of medical images by a significant amount, without losing clinically important diagnostic information. The majority of the compression techniques reported in the literature use either manual or traditional segmentation techniques to extract the informative parts of the images. The methods based upon non-uniform compression require accurate extraction of the informative part of the image to achieve higher compression rate.

**Methods:**

This research proposes unsupervised machine learning modified fuzzy c-means (FCM) clustering-based segmentation for accurate extraction of informative parts of MR images. The spatial constraints of the images are extracted using an automated region-growing algorithm and incorporated into the objective function of FCM clustering (RG-FCM) to enhance the performance of the segmentation process even in the presence of noise. Further, informative and background parts are subjected to two separate series of encoders, with higher bit rates for the informative part of the image.

**Results:**

Empirical analysis was done on the Magnetic Resonance Imaging (MRI)dataset, and experimental results indicate that the proposed technique outperforms similar existing techniques in terms of segmentation and compression metrics.

**Conclusion:**

This integration of different segmentation techniques exhibits improvement in Jaccard and dice indexes, and cascade of different encoders endorse the superior performance of the proposed compression technique. The proposed technique can help in achieving higher compression of medical images without compromising clinically significant information.

## 1 INTRODUCTION

In every aspect of human existence, images are the most important information conveyer. Due to expansion in the use of digital cameras, images are being produced continually over time, and the internet has made transmission of images from one place to another in a reliable manner thereby facilitating the storage of images for extended periods [1]. Images in their raw form can take up a lot of space in memory and, therefore, need a large bandwidth for transmission. The images generated by digital cameras or other devices contain a lot of redundancy, which is basically due to the set of correlated pixels within particular regions of the image [2]. Therefore, image compression is applied to reduce or eliminate this redundancy. This process reduces the size of the file by a significant amount, thereby enabling the storage of more photos in a given quantity of memory or disc space. Therefore, many fields of real-world applications have embraced image compression, which is now a most active field of research among several real-world applications [3].

The majority of clinical tests were earlier conducted using radiological films, but nowadays the use of digital imaging has expanded drastically for the same [4]. Many hospitals use different types of software for maintaining health records and keeping track of the patient’s clinical data, including personal information about the patient, laboratory results, digital radiological images, and medication prescriptions for patient care [5]. Medical care facilities and hospitals produce a large number of medical images of different modalities and radiological scans, such as computed tomography (CT) scans and MRI images, which must be saved in the Hospital Information System (HIS) for future use. Therefore, efficient medical image compression is required so that these images can be transferred to any person or a place within limited storage/bandwidth [6]. Many compression techniques have been introduced to restrict the need for bandwidth for transmission of medical images. These compression algorithms are mainly classified into two types labeled as lossless compression and lossy compression. In lossless compression methods, the reconstructed image after processing is similar to the input image, which means there is no information loss during the compression of the image, but the compression rate achieved is not good enough.In lossy compression techniques, the compression rate is much higher, but the quality of the image after reconstruction deteriorates because of some information loss, which may harm the patient’s diagnosis [7].

In different medical modalities, some part has the diagnostically essential information, or we can say the informative part of the image, and compressing the whole image with the same quality can lead to loss in the informative part when it is compressed with lossy techniques. Therefore, non-uniform compression can be applied to preserve the informative part because radiologists or doctors need a small portion of the image for the diagnosis, said to be the object of interest or informative part. So, the goal is to optimize the image compression such that the informative part of the image is allowed to have a greater compression quality with a low compression rate whereas the background (BG)or non-informative part is subjected to a high compression rate. Therefore, hybrid compression techniques could outperform conventional lossy and lossless techniques in terms of compression parameters. The majority of the existing compression techniques reported in the literature, for various medical modalities, either use manual or traditional segmentation techniques to extract the informative parts of the images [7-10]. Automated segmentation techniques are preferred over manual techniques to extract informative parts efficiently in the different modalities before processing the images for a higher compression ratio [11].

The objective of this research is to develop an efficient automatic segmentation technique and effective hybrid coding for improved compression of different medical modalities. The proposed work emphasizes firstly the extraction of the informative part and then non-uniform compression is applied to a segmented image, where the informative part of the image is compressed at a high bit per pixel while the remaining part is compressed at a low bit per pixel, thereby achieving overall higher compression rate (CR) with acceptable visual quality.

The contribution of this work can be summarized as follows:

- Accurate demarcation of informative parts of an image.
- Employing a cascade of lossless compression methods to achieve a higher compression rate.
- Optimization of bit rate for compression methods separately for informative and non-informative parts.

## 2 LITERATURE REVIEW

This review provides an insight into image compression techniques based on lossless, lossy, and hybrid techniques that have been reported in the existing literature.

Ramesh *et al*. [12] described a prediction of the decomposition of wavelets for medical image compression. The prediction equation depends upon the correlation analysis of each sub-band. Experimental results of this work yield a better compression ratio when compared with other existing techniques like SPHIT and JPEG2000.

Al-Faris *et al*. [13] introduced an automated extraction of the object of interest in medical images by modifying region-growing algorithms. This method introduced the segmentation using the active contours followed by morphological thinning. The proposed method was tested on a dataset of 40 images which showed improvement in parameters like relative overlap and misclassification rate.

Juliet *et al*. [14] presented the compression technique for medical images using discrete radon transform (DRT) coefficients further encoded by SPIHT algorithm. This method of incorporating DRT into SPIHT performs better than standard compression methods in terms of PSNR and CR.

Zuo *et al*. [15] presented an IMIC-ROI approach where IMIC stands for improvement in medical image compression using Region of interest (ROI) . This technique extracts the informative part of the image and lossless compression is applied to this part and lossy wavelet-based compression is applied to the background region. This approach achieves better compression results as compared to JPEG, JPEG-LS, CALIC, and ACIC-ROI.

Vaishnav *et al*. [16] introduced a hybrid technique of lossy and lossless methods to compress the medical images. This approach is based on dual-tree decomposition of wavelets cascaded with a lossless arithmetic encoder thereby achieving a better compression ratio and preserving the image quality as compared to the other conventional algorithms.

Anusuya *et al*. [17] described a lossless image compression technique to compress the Magnetic resonance imaging data using stationary wavelet transform (SWT). This method achieves better results while using minimum computational resources.

Srinivasan *et al*. [18] described a coding algorithm for efficient compression of the EEG signal matrix in which a lossy SPIHT encoder is cascaded with a lossless arithmetic encoder. This two-stage compression technique proved to be effective and showed significant improvement in compression ratio.

Suresh *et al*. [19] considered a noisy image and suggested an automatic extraction of regions of interest to detect the informative or diagnostic regions in different modalities like MRI and computed tomography (CT) images. This approach performed histogram decomposition, and statistical moments were used to estimate an automatic threshold value for the extraction of the region of interest.

Nithila *et al*. [20] reported a technique using Fuzzy C-Means clustering incorporated to region-based active contours to extract the abnormalities in lung nodules. Experimental results showed the effectiveness of this technique as there is a significant increase in similarity measures and a decrease in error rate.

Sreenivasulu *et al*. [21] suggested a region-growing technique for segmentation to extract the informative part. After this, the informative part of the image is compressed using DWT and Huffman encoder while on the other side; the background is compressed using the SPIHT algorithm. This approach yields better results in terms of segmentation and compression parameters.

Sran *et al*. [22] described the ROI based algorithm which is executed in two stages. In its first stage, the saliency model is integrated with FCM clustering for extraction of informative parts. In the second stage, the coding of the informative and background part is executed using parallel processing of non-uniform compression. The mathematical analysis showed a significant improvement in the values of CR and PSNR.

Sran *et al*. [23] introduced an automated segmented technique to detect the significant pathological regions in magnetic resonance images. This technique carried out the fast processing of the image and achieves accurate extraction of significant areas using a saliency model integrated with fuzzy thresholding. The performance of the presented method was compared with different FCM models to establish its superiority.

Sran *et al*. [24] presented visual saliency models to detect the ROI from the medical images comprising tumors. This work presented an overview of the saliency model and its technical aspects. The different saliency models like Itti-Koch, frequency synchronization, and graph-based were applied to the images but found to be less inefficient in extracting objects of interest except for the Fuzzy thresholding model.

Suma [25] described an integrated approach by cascading Discrete wavelet transform (DWT) with BPNN to compress the different images. Their approach could yield a better compression ratio (CR) while preserving the visual image quality. Experimental results obtained from this method were compared with different conventional techniques like DWT and BPNN in terms of compression ratio and PSNR.

Wei *et al*. [26] introduced refinement in the interval type-2 probabilistic fuzzy-C-means algorithm (IT2PFCM). The presented technique added adaptive spatial constraints to provide spatial information to reduce the sensitivity of the algorithm to noise. The presented method was performed on synthetic and natural images, and mathematical evaluation showed that it performs better than state-of-the-art methods.

After a review of some representative papers from the literature, the following inferences are being drawn about key issues and challenges still prevailing in the field of medical image compression:

- The compression algorithms so far reported in the literature may offer a good compression ratio but with a trade-off with some other performance parameters.
- Some of compression algorithms are effective in terms of compression parameters, but the quality of the reconstructed image is poor. It is found that the image’s domain transformation techniques can preserve the image quality while using the wavelet transform.
- Manual segmentation leads to loss in the informative part of the image because of inaccurate marking. Therefore, automated segmentation techniques should be preferred over manual techniques to avoid any kind of discrepancy in the detection of actual informative parts.
- The spatial constraints can be incorporated into clustering techniques for the extraction of informative parts in noisy images.
- Hybrid techniques using segmentation and non-uniform compression in medical images seem to be effective in the reliable transmission of images for a given bit rate. This requires in-depth investigation for more accurate extraction of the object of interest and hybrid compression algorithms to enhance the efficient transmission and storage of images.

## 3 PROPOSED METHODOLOGY

To achieve a high compression ratio, this paper proposes a technique for extraction of the object of interest in the spatial domain using proposed segmentation, and then non-uniform compression of segmented parts in the transform domain by a series of two encoders. Here, segmentation is being made more accurate to ensure a higher compression ratio. To achieve better segmentation, a region-growing-based unsupervised fuzzy c-means (FCM) clustering method is applied to the image for finer extraction of the objects of interest.

After segmentation, non-uniform compression is applied to the segments of the image, where the informative part is subjected to compression with a higher bit rate, and the background part is subjected to compression with a lower bit rate as shown in Fig. (**1**). The bit rate is tuned in such a way as to achieve a higher overall compression ratio while preserving the quality of the image.

### 3.1 Segmentation

Segmentation holds a crucial position in the area of image compression. In MRI images, the informative parts are of uneven shapes with sharp contours and irregular boundaries. The accurate segmentation requires that these uneven shapes be traced smoothly. A medical image like MRI has to detect white matter, grey matter, and other pathological tissues like tumors. The location and size of abnormal tissues in the medical modalities extracted using manual segmentation techniques are not comparable with modern-day’s fast and efficient computing algorithms. Therefore, computer-aided detecting techniques facilitate the extraction of the abnormal growth of tissues or tumors in different medical modalities, which is primarily motivated by the necessity of preserving the quality of objects of interest during the transmission of the images.

Based on similarity measures, image segmentation techniques can be classified into threshold-based, region-based, and clustering-based segmentation techniques. The similarity measures may include texture detail and other spatial properties of the image. The different thresholding methods are frequently used for image segmentation, where the threshold value may be set manually or made adaptive to enable good classification of pixels into desired categories. Still, these methods generate two classes of pixels but fail to deal with multichannel images. The region-based segmentation provides spatial information and works on the principle of similarity. Here, depending on some characteristics of the image, a coherent region is defined where all pixel values of that region are homogeneous.

Clustering-based segmentation is an unsupervised machine learning algorithm that uses the available datasets to train itself. This approach is based on the distance measure where data points which are near to each other are grouped in one cluster. In hard clustering methods like K-means clustering, a data point either belongs or does not belong to a cluster. In soft clustering methods like fuzzy-c-means (FCM) clustering, each data point belongs to a cluster with a certain probability known as membership value. FCM is found to be an effective and concise segmentation technique in many applications.

### 3.2 Proposed Segmentation Method

The selection accuracy of the object of interest depends upon the effectiveness of the segmentation technique, which can improve the compression rate while processing the different brain MRI images. FCM clustering is an effective segmentation technique to extract the region of interest, but it fails to consider spatial or feature information in the case of noisy images. Therefore, the region-growing algorithm is incorporated into the objective function of traditional FCM clustering to obtain the spatial characteristics of the target image. The region-based segmentation is useful in dealing with large amounts of data with reduced complexity.

Region-growing algorithm works on the principle of similarity and starts by picking a pixel inside the object of interest as a starting point or seed point and then allows the region to grow. This region around the initial seed grows by including adjacent pixels based on some similarity measures. This process is repeated until the difference between pixel intensity and the mean value of the region becomes larger than a certain threshold. It is found that accurate segmentation largely depends on the correct selection of seed points. Therefore, a seed point is chosen automatically using an automated threshold technique known as Otsu’s threshold. The threshold value obtained by Otsu’s algorithm separates the target image into black and white regions, and the mean pixel value of the foreground region obtained from the threshold technique is selected as the seed point.

This region-growing algorithm may not produce accurate segmentation results when there is no significant difference between the pixel values of the object of interest and background region and may require some morphological operations to extract the accurate informative parts. Moreover, the Fuzzy c-means clustering method works efficiently on noise-free images but results in inaccurate extraction of the object of interest in noisy images. Most of the medical images used for processing may not be noise-free. To address this problem, spatial constraints obtained from the modified region-growing algorithm are incorporated into the objective function of the FCM algorithm.

#### 3.2.1 Automatic Threshold using Otsu’s Thresholding

Otsu threshold determines the threshold automatically and is effective for bimodal images (images having two peaks in the histogram due to different intensity levels of the foreground and background parts of the image). This method gives a single intensity value, which is considered a threshold value for the separation of foreground and background pixels. The mean pixel intensity of the foreground image is chosen as the seed point of the region-growing method.

#### 3.2.2 Extraction of Spatial Information using Region-Growing Algorithm

The region-growing algorithm can extract the defects and provide the spatial information of the informative parts. The region around the seed point continues to grow by including neighboring pixels until the intensity difference is within the given threshold and then converges when no further merging is possible.

#### 3.2.3 ROI Extraction by Incorporating Region-Growing Algorithm into FCM Clustering

FCM is the unsupervised machine learning algorithm based on the distance measure, where two or more clusters are formed by grouping data points that are close to each other. The segmented image obtained from the region-growing algorithm possesses similar feature values and highly correlated neighboring pixels. The spatial information is incorporated into the objective function of standard fuzzy c-means clustering for splitting the data into different clusters. The distance of a new data point from the centroid (mean value of cluster) is calculated using Euclidean distance and membership values (probability of data point belonging to a cluster), then the values of centroid and membership are updated. This algorithm goes through several iterations until the optimum value of the objective function is achieved. The objective function of FCM clustering for splitting the data obtained from the region-growing algorithm 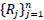 into *m* different clusters is given by:

**Table:** 

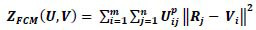	(1)

Where *U_ij_* is the degree of membership of data point *R_j_* in the cluster *V_i_*, *m* is the number of clusters, n is the number of data points, *R_j_* is the pixel value of a particular data point, *V_i_* represents the centroid of the cluster and p is the weight exponent.

**Figure:**
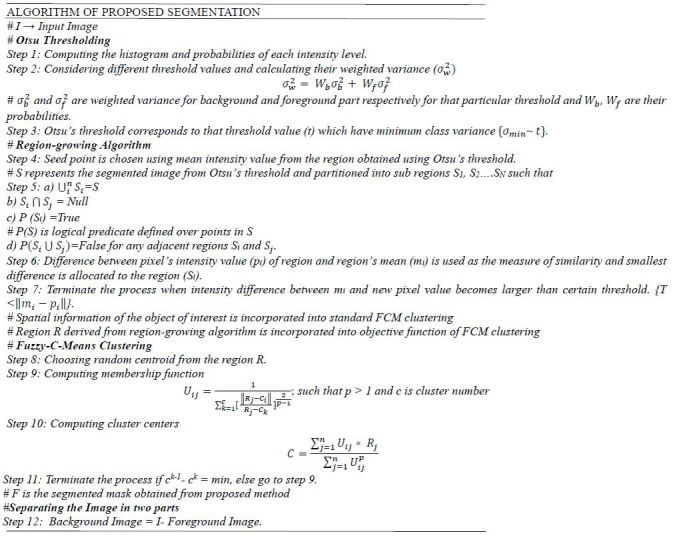


#### 3.2.4 Segmentation Performance Parameters

The accuracy of the segmentation is evaluated using the Jaccard similarity index (J), dice coefficient (D)and percentage of foreground area.

• **Jaccard Index** is used for measuring the similarity and diversity of sample sets (R, G), and it is given by:

**Table:** 

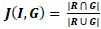	(2)

• **Dice similarity coefficient** is the spatial overlap index, which measures the degree of overlap of a given set with ground truth set. It is given by:

**Table:** 

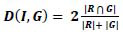	(3)

Where R is the segmented informative part, and G is the ground truth (GT) image.

• **Foreground Area calculation** is the percentage of area extracted from the proposed segmentation method. It is given by:

**Table:** 

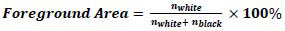	(4)

The proposed segmentation method (RG-FCM) and standard FCM clusteringare tested on the different MRI dataset (available online at https://www.kaggle.com/datasets/navoneel /brain-mri-images-for-brain-tumor detection) [27] to compare the effectiveness of the proposed approach, on the basis of above-mentioned parameters. And the results so obtained are being summarized in Table **1**.

For better visualization of performance comparison, different values of Jaccard index, dice similarity index and the percentage of foreground area are graphically presented in Figs. (**2**-**4**).

From these Tables and Figures, it can be observed that the efficiency of the proposed segmentation technique RG-FCM is far better than the standard FCM method. Also, the percentage of foreground area is closest to the ground truth (GT) values for all images, which is an indication of a more accurate estimation of the area of interest. In other words, the smaller the area of the foreground part, the higher the compression rate; because it is the foreground part of the image that is encoded with a higher bit rate. Therefore, it is validated that the incorporation of spatial information into FCM clustering leads to more accurate segmentation of the given image. Now it can be expected that the improvement achieved in segmentation will further enhance the efficiency of the compression algorithm.

#### 3.2.5 Statistical Analysis

The extraction accuracy of the object of interest is evaluated using the Jaccard Index and Dice Index, whereas Precision, Recall, and F-measure are statistical measures for boundary-based evaluation of the segmentation technique. The comparative analysis of execution time is also carried out to evaluate the computational complexity of the proposed technique. Table **2** exhibits the statistical performance of various segmentation metrics of RG-FCM and FCM.

The statistical analysis depicted in the above table justifies the accurate demarcation of the object of interest using RG-FCM. The improved values of Precision, Recall, and F-measure using RG-FCM indicate that the proposed algorithm is efficient in extracting diagnostic information with smooth boundaries and proved to be robust in noisy images. The improved values of Jaccard and dice indexes justify that the proposed segmentation technique can yield accurate demarcation of the object of interest. In terms of time complexity also, the proposed approach outperforms standard FCM where mean execution time is reduced by 5.5 seconds. Therefore, the proposed segmentation technique can meet the high computational demand in clinical applications. The statistical analysis of segmentation metrics illustrated in Fig. (**5**) shows the superiority of the proposed method.

For qualitative analysis of segmentation results, images obtained from proposed RG-FCM and standard FCM are shown in Fig. (**6**).

From this figure, one can make out that the background and foreground parts obtained from the proposed segmentation method are almost similar to ground truth images. These segmented images are further processed in parallel and subjected to high compression rates and low compression rates, respectively. The accuracy of the segmentation can lead to an overall high compression rate without losing essential details of transmitted images.

### 3.3 Compression

The segmented image is subjected to Fourier transform to split the high-frequency and low-frequency coefficients. Sometimes, it is observed that the information associated with particular frequencies may not be sufficiently represented. Therefore, a multi-resolution method is required that can allow faithful transmission of images in frequency and spatial domains. The wavelet transform is applied here as a multi-resolution tool, where different windows are used to get good resolution in time and frequency. The continuous wavelet transforms (CWT) are time-consuming due to their complex integration at each particular time, whereas DWT can be implemented through sub-band coding. The latter can remove block artifacts and aliasing effect which were the main causes of distortion of information in the Fourier transform. Therefore, the DWT-based Set Partitioning in Hierarchical Tree (SPIHT) compression technique is used in the proposed algorithm. SPIHT is embedded in DWT as it has low computational complexity, offers a good compression rate, and finally generates a compressed bitstream at the output of the encoder. This bitstream is further incorporated into an efficient lossless encoder (Huffman encoder) to exploit the redundancy of the bitstream obtained at the output of the SPIHT encoder, thereby enabling a further increase in the compression ratio without losing important information.

Therefore, segmented images are subjected to discrete wavelet transform (DWT) to get their transform domain representations before compression. In this domain only, a series of compression methods are applied at selective bit rates. Now compression at different bit rates is achieved by cascading two encoders, as shown in Fig. (**7**).

Firstly, the SPIHT encoder partitions the decomposed wavelet coefficients into significant and non-significant pixels and performs the compression on the wavelet coefficients. The desired compression can be achieved by controlling the bit rate of the algorithm for both types of pixels. SPIHT is being used as a lossy encoder (less lossy on the object of interest and more lossy on the background part), which may cause some losses during transmission. Therefore, the region of interest is processed at a high bit rate to preserve the image quality, and the region of background part (non-significant) is processed at low bit rates to achieve an overall high compression rate. Further, the output bit stream of the SPIHT encoder is processed by a lossless Huffman encoder to exploit the redundancy present in the bit stream at the output of the SPIHT encoder, to further increase the compression ratio.

#### 3.3.1 Discrete Wavelet Transform

The wavelet transform represents the image as a sum of wavelet functions with different locations and scales, exhibiting the least correlation. The wavelet transform allows good localization in frequency and space, in which high frequency corresponds to horizontal, vertical, and diagonal details while low frequency corresponds to approximation details of the image. The number of decomposition levels isgiven by:

**Table:** 

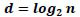	(5)

where *n* is the number of elements in a row.

Then a bi-orthogonal wavelet filter (‘***bior* 4.4**’) is chosen to avoid any overlapping in approximation and detail functions. This wavelet filter produces different frequency sub-bands (LL, LH, HL and HH), where ‘L’ stands for low frequency and ‘H’ stands for high frequency. The LL sub-band has approximation details which are comprised of a large amount of information of the segmented image.

#### 3.3.2 SPIHT Encoder

After transforming the image into wavelet coefficients, SPIHT encoder is employed to carry out the following processing:

• To partition the coefficients into significant and insignificant coefficients using the following threshold function:

**Table:** 

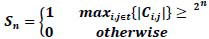	(6)

Where n is log_2_ *Cmax*, *S_n_* is a significant set of coordinates, and *C_ij_* is the value of the coefficient at a particular coordinate (*i,j*).

• After this, it makes use of: sorting pass and refinement pass. During sorting pass, all pixels are categorized in three sets: list of insignificant pixels (LIP), which consists of those coefficients having less magnitude than threshold; list of insignificant sets (LIS), consisting of coefficients determined by structures of trees and have lesser magnitude than the threshold; list of significant pixels (LSP), consisting of coefficients having greater magnitude than threshold [21]. The maximum bits required by the largest coefficient in its spatial tree representation are given by *b_max_*:

**Table:** 

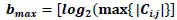	(7)

• During the refinement pass, coefficients in the LIP are tested using the threshold to determine whether these are significant or not. Out of these, the significant coefficients are transferred to the LSP, including their sign bit. Now the sets present in the LIS are checked to see whether they are significant or not, and if that particular set is significant, then it will be divided into subsets. Further, those subsets that have only one coefficient, and if found to be significant, then it will be transferred to LSP, and if it is insignificant, then it will be transferred to LIP.

• Now the final output of the refinement pass will be the most significant bit (MSB) of the coefficients in the LSP. Further, the value of *n* is decremented and the threshold value is reduced from the previous one, and the same process of sorting pass and refinement pass will continue.

These passes will continue until either the desired or selected compression rate is attained or *n*=0, and all nodes in the LSP have all their bit output. The case of *n*=0 will result in almost the same reconstruction as of the input giving almost lossless compression, but the compression rate will be less. For the higher value of the threshold, some coefficients will be discarded, which results in lossy compression. Therefore, the desired compression can be achieved by controlling the bit rate of the SPIHT algorithm [22].

#### 3.3.3 Huffman Encoder

An output bit stream of SPIHT encoder is further sent to the Huffman encoder to exploit the redundancy present in this bit stream. It starts with calculating the probability of every symbol present in the bit stream. The probability of each symbol is arranged in descending order, and these symbols are coded individually, then the symbols with the lowest probabilities are merged to get the final code. This repetitive process of merging the symbols is carried out until only two probabilities of two compound symbols are left. As a result, a code in tree form is generated, and by labeling this code tree, Huffman codes are acquired. Finally, the branch digits are read in sequence from the root node of the tree to each terminal node to get the final code [28].

**Figure:**
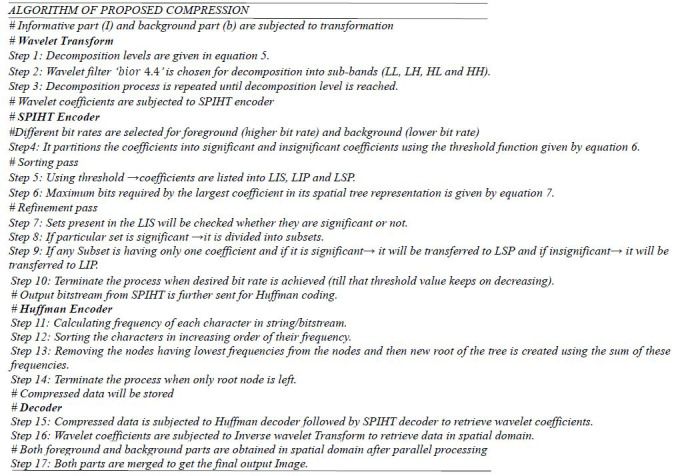


The final compressed bit stream obtained from the Huffman encoder is transmitted or stored. For reconstruction of original image, this compressed file is decoded using the Huffman decoder followed by the SPIHT decoder, resulting in wavelet coefficients. These coefficients are finally reverted back into the original data or image using the Inverse Wavelet Transform at the receiver end. The decoding sequence of the proposed compression technique is shown in Fig. (**8**).

The output data is obtained in the spatial domain from parallel processing of segmented images at different bit rates. The object of interest is processed at high bit rates to retain the good quality of the informative part, and the background part is processed at a low bit rate to achieve a high compression rate. These images are fused to get a final output image, then several performance parameters, regarding original image, are calculated for quantitative evaluation to prove the effectiveness of the proposed technique.

#### 3.2.4 Compression Performance Parameters

The parameters used to assess the proposed algorithm's effectiveness are bits per pixel (BPP), peak signal-to-noise ratio (PSNR), mean square error (MSE), compression ratio (CR), and structural similarity index (SSIM), which are being defined below:

• **MSE** is the standard of digital image quality index, and high values of mean square error imply low-quality digital image, and its mathematical expression is given by:

**Table:** 

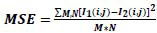	(8)

where *I_1_* is the input and *I_2_* is the output image, and (*i, j*) are rows and columns, respectively.

• **SSIM** measures the loss of structural content of the image caused by the processing of the image, and the mathematical expression for SSIM at (x, y) coordinates is:

**Table:** 

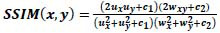	(9)

where *u* is the average and *w* is the variance of respective (*x, y*) coordinates.

• **PSNR** is the ratio of the maximum value of a signal to the power of distorting noise, and the mathematical expression for PSNR is given by:

**Table:** 

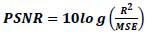	(10)

where *R* is the maximum fluctuation, and different values are assigned to *R* for different

data.

• **CR** is the ratio of the size of the original image to the size of the compressed image or bit stream. The proposed method calculates the compression ratio of the informative part and background part independently. Finally, both parts of the image are processed in successive order after performing compression. The full image, region of interest (ROI), and non-region of interest (non-ROI) are represented as *I_full_*, *I_r_* and *I_nr_* respectively. Encoded bytes for non-ROI and ROI are referred to as *E_nr_* and *E_r_* respectively.

**Table:** 

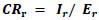	(11)

**Table:** 

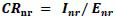	(12)

**Table:** 

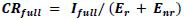	(13)

where *CR_r_* is the compression ratio of the ROI or informative part, *CR_nr_* is the compression ratio of the non-ROI part or background, and *CR_full_* is the compression ratio of the full image.

• **Bit Per Pixel** is the number of bits used for coding the pixel value. A higher bit rate will provide superior quality of the image, and similarly, BPP (bit per pixel) of ROI, non-ROI, and the full image is calculated using their respective encoded bytes as described below:

**Table:** 

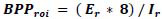	(14)

**Table:** 

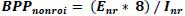	(15)

**Table:** 

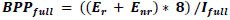	(16)

The mathematical evaluation of employing a series of encoders using hybrid segmentation is carried out using the above parameters and results of the proposed compression technique are compared with the standard existing compression algorithms like JPEG, JPEG 2000, and SPIHT.

JPEG is an image compression standard based on discrete cosine transform (DCT) for transforming images from the spatial domain (pixel values) to frequency coefficients, and after zigzag scanning and quantization of coefficients, a lossless encoder is cascaded with it to achieve compression, but variations in the overall characteristics from block-to-block give gradations in quantization, and during the reconstruction of the image, different blocks can be identified known as blocking artifacts. These artifacts may lead to blurriness in the reconstructed images when images are processed at low bit rates.

Therefore, JPEG is not preferable at very low bit rates, while on the other hand, JPEG 2000 uses two different wavelet transforms, the coefficients which are scalar quantized to yield integers. then encoder encodes the bits of all quantized coefficients of a code block, starting with the most significant bits and progressing to less significant bits. Therefore, JPEG 2000 generally provides a high PSNR of the reconstructed images compared to JPEG, but the computational complexity of the JPEG 2000 standard is higher due to the use of different wavelet transformation techniques. SPIHT generally uses wavelet decomposition and further performs partitions to achieve the compression as explained in the proposed methodology, SPIHT is proven to be more robust than standard image compression standards JPEG and JPEG 2000 in terms of the quality of reconstructed images and uses of the single transformation technique.

The analysis of the proposed method's numerical results is presented in this section. BPP, CR, MSE, PSNR, and SSIM are the parameters used to evaluate the effectiveness of this technique, and its experimental results are compared with different similar existing image compression techniques like JPEG, JPEG2000, and SPIHT. These techniques are tested on the same dataset of brain MRI images to evaluate the efficiency of the proposed technique. The performance analysis of the proposed approach for ROI part, non-ROI part, and full reconstructed image at different bit rates is shown in Tables **3** and **4**.

The bit rate for the proposed technique is varied from 0.50 bpp (bit per pixel) to 1.00 bpp for the informative part and 0.25 bpp (bit per pixel) to 0.50 bpp for the background part to achieve non-uniform compression. More than 50 medical images present in the dataset have been subjected to the proposed method to ascertain its versatility. Fig. (**9**) illustrates the statistical analysis of CR, MSE, SSIM, and PSNR for the informative parts and the entire images.

Investigation of these graphs and tables reveals that high bit rates can achieve a good-quality of the image, and low bit rates result in the deteriorated quality of the image. Therefore, the informative part of the image is processed at high bit rates, which helps to retain the informative part of good quality (high PSNR and SSIM of the informative part), and the background is processed at low bit rates to achieve a high compression rate. The compression rates of the entire image depend upon the bit rates and area of the extracted informative parts. The coding of an image with a small object of interest results in a high compression rate, and a large object of interest results in low compression rates, as depicted in the Table **5**.

Thus, the small size of the object of interest results in high compression rates, and an overall compression rate of 75.86 is achieved with good visual quality of the informative part. This efficient transmission and storage of the object of interest can be useful for patients in other remote locations.

The mathematical results of the proposed compression algorithm are compared with standard existing techniques like SPIHT, JPEG, and JPEG2000. The statistical performance (mean of the dataset) of the proposed method at different bit rates is being compared with the average performance of standard methods, as summarized in Tables **6**-**8**.

The investigation of the above tables reveals that the proposed method achieves a higher CR at a given bit rate. It also achieves higher values for PSNR and SSIM, thereby ensuring the better visual quality of the reconstructed image. The graphical representation of the performance comparison of PSNR and CR at different bit rates is demonstrated in Figs. (**10** and **11**) for quick visualization of the results. This graphical analysis also validates the effectiveness of the proposed compression technique.

Fig. (**11**) shows that CR of the informative part of the proposed method is higher than standard SPIHT due to the addition of a lossless encoder at the same bit rate, and CR of the full image is far better than standard SPIHT as the background part is compressed at lower bit rates. Therefore, higher compression rates are achieved using the proposed method.

It is a well-known fact that the quality of a reconstructed image can be improved by increasing the bit rate, but at the cost of reduced values of CR. But the proposed method can give higher CR at a higher bit rate when compared with other methods. This improvement in CR without compromising the image quality of the informative part can be attributed to the accurate segmentation of the source image and the use of cascades of two encoders. This quantitative analysis can establish the superior performance of the proposed approach, which is further substantiated by qualitative analysis of the reconstructed image from the proposed approach as depicted in Fig. (**12**).

The visual quality of the reconstructed image, obtained from non-uniform compression, is compared for different combinations of bit rates. The visual inspection of these images reveals that a higher CR can be obtained with an acceptable-quality of recovered image. Overall, the visual quality of reconstructed images endorses the superior performance of the proposed method.

#### 3.2.6 Quantitative analysis using Confidence Intervals

Confidence interval (CI) is a statistical measure that estimates variations in values of different parameters within an expected range. The minimum range of confidence interval ensures the reproducibility of the experiment. The confidence interval is estimated based on a desired confidence level. CI is calculated as:

**Table:** 

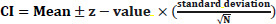	(17)

For confidence levels of 90%, 95%, and 99%, the z value is 1.65, 1.96, and 2.58, respectively, and N is the number of images from the dataset. Confidence intervals are constructed at different confidence levels for different segmentation metrics (Jaccard Index & dice index) and compression metrics (Compression Ratio & Peak signal-to-noise ratio), the values of which are summarized in the Table **9** .

These Confidence Intervals provide the estimate of the range of values (lower limit-upper limit) in which the values of different parameters may fall. From the table, it can be observed that the range for the different parameters varies with confidence levels, and it gets wider as confidence level increases. This quantitative analysis demonstrates that the Jaccard Index varies from 0.9539 to 0.9741 at 95% confidence level for different images, and similarly Dice Index varies from 0.9651 to 0.9869. The confidence intervals for compression ratio and PSNR of the foreground part at bit per pixel 1.00 (FG) & 0.5 (BG) are constructed at different confidence levels, and the range of values gets wider with an increase in confidence levels. The compression ratio varies from 35.20 to 42.06, and PSNR of the object of interest varies from 38.16 to 43.86 at 95% confidence level for different images.

## 4 RESULT AND DISCUSSION

The incorporation of the spatial constraints obtained from the modified region-growing method into fuzzy c-means clustering results in the accurate extraction of objects of interest in magnetic resonance images, and this refined extraction of informative parts leads to high compression rates. The different medical imaging modalities such as computed tomography scans and ultrasound scans can employ the proposed algorithm, expanding its applications to various clinical implications. Further, soft computing techniques like fuzzy clustering can be incorporated to extract different spatial constraints to enhance the segmentation accuracy of ROI part.

### 4.1 Implications for Medical Applications

The rate at which medical images are being generated is increasing exponentially, which enforces the necessity of more storage and more bandwidth for transmission. Therefore, an accurate demarcation of the object of interest is required for efficient compression of different medical images to reduce the transmission bandwidth and storage space. Segmentation-based compression techniques play a key role in biomedical applications that enable doctors to improve the accuracy of the diagnostic process. The proposed technique provides an alternative concept for the staff working in the medical field. The proposed segmentation-based compression technique can efficiently be employed in different medical imaging modalities for high compression rates while preserving the quality of the diagnostically significant information. This efficient transmission and storage of medical images can be expanded to clinical use for patients in other remote locations.

### 4.2 Addressing Issues of Real Time Processing and Different Modalities

A huge number of medical images with variant modalities like PET scans and MR images are required to be preserved in Hospital Information Systems (HIS) for future references, and storage of these images is posing a great challenge due to availability of limited space, and difficult to employ limited bandwidth to transmit large size images for different opinions and consultations. With the increasing utilization of medical imaging in clinical practice and large data generated by various modalities, the storage and transmission of large datasets require medical image compression. The proposed method can be employed in real-time as it has low execution time in extracting essential details and gives good results in a both qualitative and quantitative manner. This method can efficiently extract objects of interest, which results in efficient compression and reconstruction of medical images to achieve fast transmission, which can effectively assist clinicians or doctors in diagnosing patients in real-time. In addition to MR images, the proposed method can be employed for different medical imaging modalities like PET scans and CT scans, as it has low run time, which ensures the fast transmission of images and yields better performance through various evaluation segmentation and compression metrics.

## CONCLUSION

MRI and CT scans have been frequently used in telemedicine. These images in their raw form can take up a lot of space in RAM and, therefore, need high bandwidth for transmission and storage of images. Hence, developing an effective hybrid compression technique using segmentation and non-uniform compression for such images without affecting the informative parts of the image is essential. Multiple performance parameters were calculated to evaluate the effectiveness and efficiency of the segmentation and compression processes of the proposed technique. Quantitative and qualitative analysis demonstrates that the proposed technique attained better segmentation and compression metrics when compared with similar existing compression methods.

## Figures and Tables

**Fig. (1):**
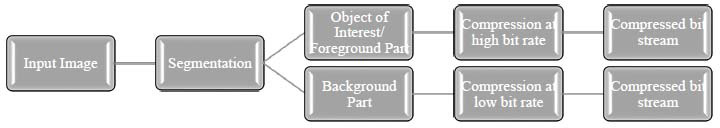
Structure of proposed compression method with bit rate selection.

**Fig. (2):**
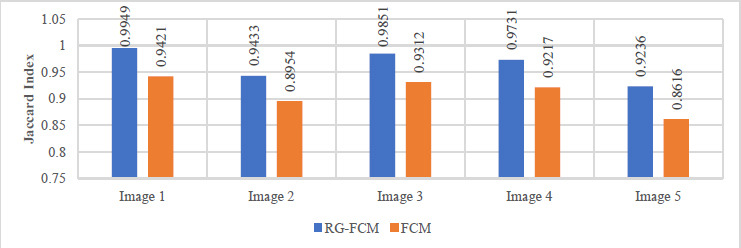
Jaccard index profile.

**Fig. (3):**
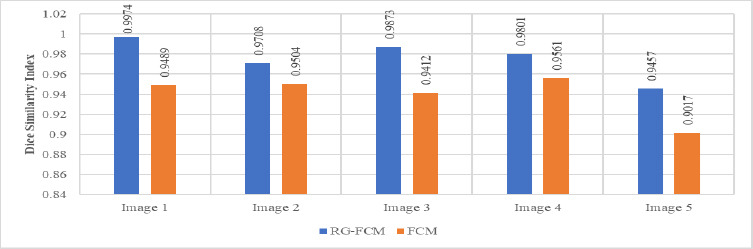
Dice index profile.

**Fig. (4):**
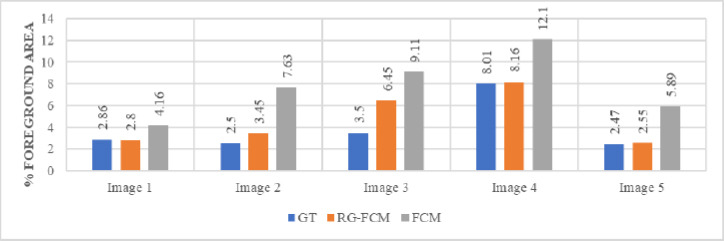
Percentage of foreground area.

**Fig. (5):**
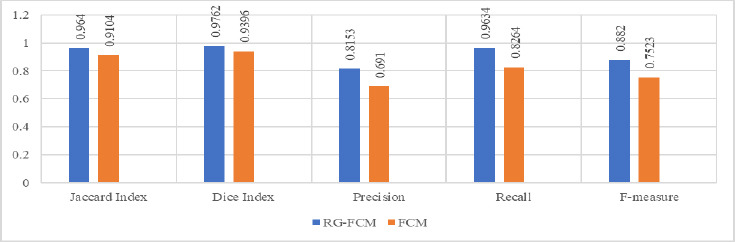
Statistical analysis based performance of segmentation methods.

**Fig. (6):**
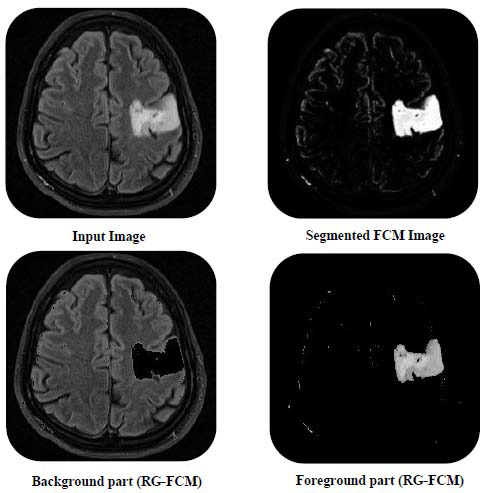
Comparison of segmented images.

**Fig. (7):**
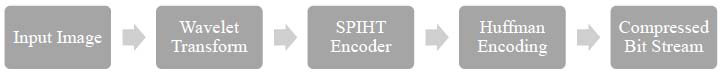
Cascading of different encoders.

**Fig. (8):**
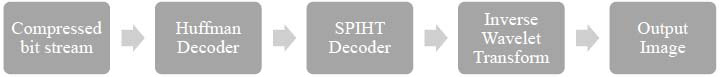
Decompression method for the proposed compression algorithm.

**Fig. (9):**
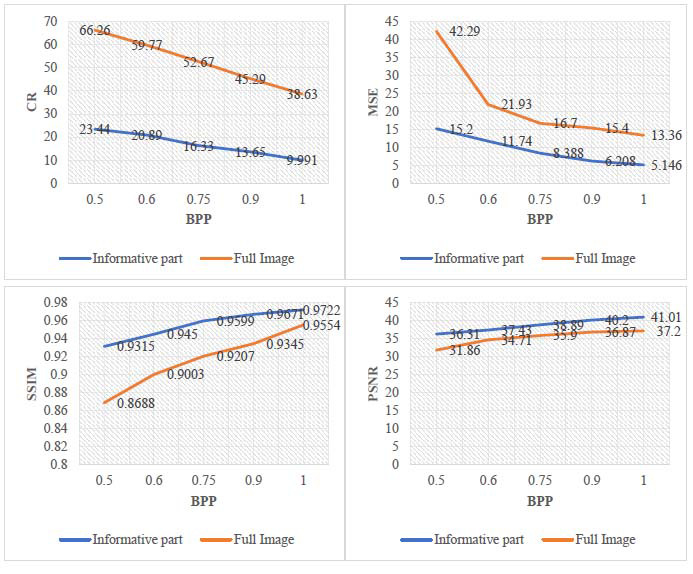
Plots of CR, MSE, SSIM, and PSNR v/s BPP for informative part and full image for the proposed method.

**Fig. (10):**
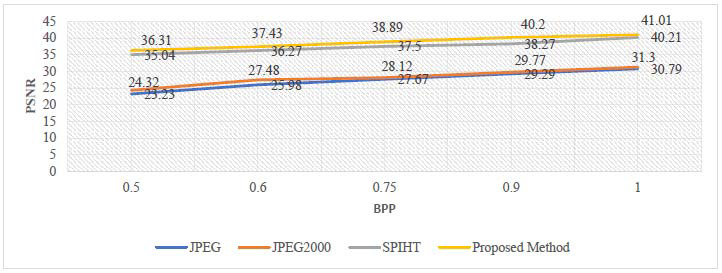
Comparison of PSNR with standard methods.

**Fig. (11):**
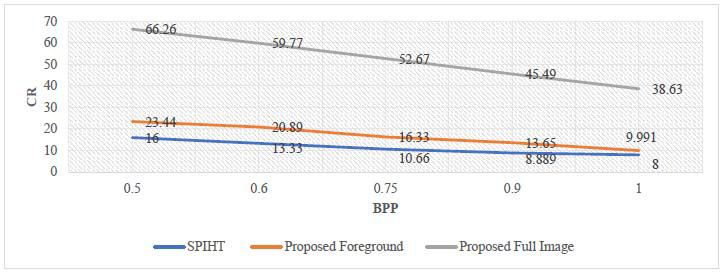
Comparison of CR of the proposed method with SPIHT.

**Fig. (12):**
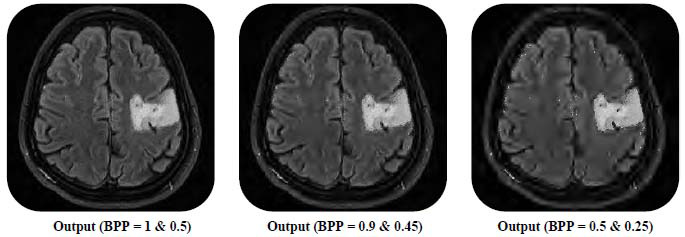
Reconstructed images of the proposed method at different bit rates.

**Table 1:** Segmentation: Performance comparison of proposed RG-FCM with Standard FCM.

**S.No.**	**-**	**Jaccard Index**		**Dice similarity coefficient**		**% Foreground Area**		
		**RG-FCM**	**FCM**	**RG-FCM**	**FCM**	**GT**	**RG-FCM**	**FCM**
1	Images 1	0.9949	0.9421	0.9974	0.9489	2.86	2.8	4.16
2	Images 2	0.9433	0.8954	0.9708	0.9504	3.13	3.45	7.63
3	Images 3	0.9851	0.9312	0.9873	0.9412	5.67	6.45	9.11
4	Images 4	0.9731	0.9217	0.9801	0.9561	8.01	8.16	12.1
5	Images 5	0.9236	0.8616	0.9457	0.9017	2.47	2.55	5.89

**Table 2:** Statistical analysis of segmentation evaluation metrics.

**Methods**	**Jaccard** **Index**		**Dice Index**		**Precision**		**Recall**		**F-measure**		**Execution Time (s)**	
Comparison	RG-FCM	FCM	RG-FCM	FCM	RG-FCM	FCM	RG-FCM	FCM	RG-FCM	FCM	RG-FCM	FCM
Mean	0.964	0.910	0.976	0.939	0.815	0.691	0.963	0.826	0.882	0.752	3.12	7.67
Standard Deviation	0.038	0.067	0.041	0.087	0.080	0.193	0.059	0.28	0.101	0.320	0.29	2.65

**Table 3:** Performance of proposed method for informative part and full image at various bit rates.

	**Informative part parameters**					**Full Image parameters**			
**S. No.**	**BPP**	**CR**	**MSE**	**PSNR**	**SSIM**	**CR**	**MSE**	**PSNR**	**SSIM**
1	0.5	23.44	15.2	36.31	0.9315	66.26	42.29	31.86	0.8688
2	0.6	20.89	11.74	37.43	0.945	59.77	21.93	34.71	0.9003
3	0.75	16.33	8.388	38.89	0.9599	52.67	16.7	35.9	0.9207
4	0.90	13.65	6.208	40.2	0.9671	45.29	15.4	36.87	0.9345
5	1.00	9.991	5.146	41.01	0.9722	38.63	13.36	37.2	0.9554

**Table 4:** Performance of proposed method for background part at different bit rates.

	**Informative part parameters**					**Full Image parameters**			
**S. No.**	**BPP**	**CR**	**MSE**	**PSNR**	**SSIM**	**CR**	**MSE**	**PSNR**	**SSIM**
1	0.25	82.14	62.42	30.17	0.8433	66.26	42.29	31.86	0.8688
2	0.3	75.45	36.19	32.54	0.8536	59.77	21.93	34.71	0.9003
3	0.4	68.86	31.69	33.12	0.8857	52.67	16.7	35.9	0.9207
4	0.45	55.75	25.99	33.98	0.9019	45.29	15.4	36.87	0.9345
5	0.5	48.86	21.69	34.86	0.9159	38.63	13.36	37.2	0.9554

**Table 5:** Variation of Compression ratio with area of foreground part.

**Image**	**% of object of interest**	**Bit Rate FG**	**BG**	**Compression Ratio Entire Image**
Image 1	2.80%	0.5	0.25	75.86
Image 2	3.45%	0.5	0.25	68.89
Image 3	6.45%	0.5	0.25	59.6
Image 4	8.67%	0.5	0.25	53.77

**Table 6:** Peak signal to noise Ratio (PSNR) comparison with standard methods.

**S. No**	**BPP**	**JPEG**	**JPEG2000**	**SPIHT**	**Proposed Method ROI Part**	**Full Imag**
1	1	30.79	31.3	40.21	41.01	37.2
2	0.9	29.29	29.77	38.27	40.2	36.86
3	0.75	27.67	28.12	37.5	38.89	35.9
4	0.6	25.98	27.48	36.27	37.43	34.71
5	0.5	23.23	24.32	35.04	36.31	31.86

**Table 7:** Structural similarity index (SSIM) comparison with standard methods.

**S. No**	**BPP**	**JPEG**	**JPEG2000**	**SPIHT**	**Proposed Method ROI Part**	**Full Imag**
1	1	0.8001	0.812	0.9543	0.9722	0.9554
2	0.9	0.7195	0.728	0.9485	0.9671	0.9345
3	0.75	0.6991	0.7077	0.9442	0.9599	0.9207
4	0.6	0.6572	0.6685	0.931	0.945	0.9003
5	0.5	0.605	0.6203	0.9146	0.9315	0.8688

**Table 8:** Compression ratio (CR) comparison with SPIHT.

**S. No**	**BPP**	**SPIHT**	**Proposed Method ROI Part**	**Full Imag**
1	1	8	9.991	38.63
2	0.9	8.889	13.65	45.29
3	0.75	10.66	16.33	52.67
4	0.6	13.33	20.89	59.77
5	0.5	16	23.44	66.26

**Table 9:** Confidence intervals for performance metrics.

** CI at different confidence levels **	Confidence Intervals constructed at 90%	Confidence Intervals constructed at 95%	Confidence Intervals constructed at 99%
** Different Parameters **
Jaccard Index	0.9555-0.9725	0.9539-0.9741	0.9506-0.9773
Dice Index	0.9668-0.9852	0.9651-0.9869	0.9617-0.9903
Compression Ratio at bit per pixel (1 & 0.5)	36.75-40.51	35.20-42.06	34.13-43.13
PSNR of object of interest at bit per pixel (1 & 0.5)	39.23-42.79	38.16-43.86	37.79-44.23

## Data Availability

54 MRI images were taken from the available online database [https://www.kaggle.com/datasets/navoneel/brain -mri-images-for-brain-tumor detection] [27] for testing the effectiveness of proposed method. This data is tested in MATLAB R2017b using the proposed algorithm.

## LIST OF ABBREVIATIONS

FCM: Fuzzy c-means
HIS: Hospital Information System
CT: Computed tomography
CR: Compression rate
ROI: Region of interest
DRT: Discrete radon transform
SWT: Stationary wavelet transform
SPIHT: Set Partitioning in Hierarchical Tree
MSB: Most significant bit
MSE: Mean square error
SSIM: Structural similarty indeix
BPP: Bits per pixel
PSNR: Peak signal-to-noise ratio
HIS: Hospital Information Systems
